# Numerical Investigation of the Influence of Ultimate-Strength Heterogeneity on Crack Propagation and Fracture Toughness in Welded Joints

**DOI:** 10.3390/ma15113814

**Published:** 2022-05-27

**Authors:** Yueqi Bi, Xiaoming Yuan, Mingrui Hao, Shuai Wang, He Xue

**Affiliations:** 1 National Engineering Laboratory for Coal Mining Machinery and Equipment, Taiyuan 030000, China; 2 Taiyuan Research Institute of China Coal Science and Engineering Group Co., Ltd., Taiyuan 030000, China; yuanxiaoming@tyccri.com (X.Y.); haomingrui@tyccri.com (M.H.); 3 School of Mechanical and Electrical Engineering, China University of Mining and Technology, Beijing 221008, China; 4 School of Mechanical Engineering, Xi’an University of Science and Technology, Xi’an 710054, China; 17101016005@stu.xust.edu.cn (S.W.); xuehe@xust.edu.cn (H.X.)

**Keywords:** crack propagation path, uneven distribution of ultimate strength, fracture toughness, user-defined field variable subroutine

## Abstract

The mechanical properties of dissimilar metal-welded joint materials are heterogeneous, which is an obstacle to the safety evaluation of key welded structures. The variation of stress–strain conditions at the crack tip caused by mismatch of material mechanical properties in dissimilar metal-welded joints is an important factor affecting crack propagation behavior. To understand the influence of uneven distribution of ultimate strength of the base metal and the welded metal on the crack propagation path, fracture toughness, as well as the mechanical field at the crack tip in the small-scale yield range, the user-defined field variable subroutine method is used to express continuous variation characteristics of welded joint ultimate strength in finite element software. In addition, the J-integral during crack propagation is calculated, and the effect of the ultimate strength on the J-integral and the stress field at the crack tip are analyzed. The results show that as the crack propagation direction is perpendicular to the direction of ultimate strength, the gradient of ultimate strength increases from |*G_y_*|= 50 to |*G_y_*|= 100 MPa/mm, the crack deflection angle increases by 0.018%, and the crack length increases by 1.46%. The fracture toughness of the material decreased slightly during crack propagation. Under the condition that the crack propagation direction is the same as the direction of ultimate strength, the crack propagation path is a straight line. As the gradient of ultimate strength increases from *G_x_* = 50 to *G_x_* = 100 MPa/mm, the crack propagation length decreases by 5.17%, and the slope of fracture toughness curve increases by 51.63%. On the contrary, as the crack propagates to the low ultimate strength side, the crack propagation resistance decreases, the ultimate strength gradient increases from *G_x_* = −100 to *G_x_* = −50 MPa/mm, and the slope of the fracture toughness curve decreases by 51.01%. It is suggested to consider the relationship between crack growth behavior and ultimate strength when designing and evaluating the structural integrity of cracks at the material interface of dissimilar metal-welded joints.

## 1. Introduction

In general, dissimilar metal-welded joints (DMWJs) in engineering structures are used to connect different metals. Due to strong heterogeneity of the microstructure and mechanical properties at the interface between two metal materials, this part is a weak link of the welded joint, which seriously affects the safety of engineering structures [1,2,3] and service life evaluation [4,5]. Therefore, it is of great significance to accurately design the integrity and evaluate the crack at the interface of bi-materials. When analyzing the defect resistance behavior of welded structures with cracks, the mismatch of material mechanical properties is an important factor that should be considered [6,7]. The research on the mismatch of material mechanical properties includes yield strength, work hardening, etc., and the strength mismatch is usually defined by a ratio of the yield strength of weld metal to base metal. Global mismatch affects the defect mechanical driving force, crack tip stress field, crack tip constraint, and crack resistance, which have been widely studied and taken into account during structural integrity evaluation [8,9,10]. The application range of the J-integral is extended from linear elastic solid to small-deformation elastic-plastic solid without unloading, so it has been widely used in elastic-plastic fracture mechanics [11,12]. Yang et al. [13] conducted an experimental study on the local fracture resistance of two cracks in the weakest area of dissimilar metal-welded joints (austenitic stainless 508 (A508) heat-affected zone crack and A508/alloy 52 Mb interface crack), and they discussed the in-plane restraint effect caused by the crack depth and its mechanism. Wang et al. [14] experimentally studied the local fracture resistance and crack propagation behavior of dissimilar metal-welded joints (DMWJs) using single-edge notched bend (SENB) specimens, as well as analyzed the effects of local strength mismatch on the local fracture resistance, crack propagation path, and integrity evaluation of DMWJs. Østby et al. [15] investigated the constraint effect of different temperatures on the near-end stress field, and they found that the change of stress depends on the strong hardening mismatch. The interfacial crack tip constraint and J-integral of bi-materials with plastic hardening mismatch are studied. However, the material ultimate strength mismatch also determines the expansion resistance and expansion driving force, and is also an important factor that should be considered. Especially for the welded joint composed of bimetals with large differences in mechanical properties, the global mismatch is difficult to accurately evaluate the safety of the weldment. Therefore, it is necessary to analyze the local ultimate strength mismatch in the metal connection area of the weldment. When the numerical simulation method is used to study the mismatch interface crack evaluation of material mechanical properties, the sandwich structure finite element model is mostly used, in which the materials with different mechanical properties are cut apart. The different homogeneous material mechanical property parameters are provided in their respective regions when establishing the finite element model [16,17,18]. For example, Fan et al. [19,20,21] established the sandwich structure finite element model and studied the local fracture resistance, and analyzed that the local material constraint effect and crack path deviation are strongly affected by the “local strength mismatch” in the bi-material interface area of dissimilar metal-welded joints, as well as constraint of the work hardening mismatch effect on the crack tip, the near-tip stress field, and the J-integral of the bi-material interface crack. Xue et al. [22,23] used the sandwich structure finite element model to analyze the effect of material yield strength mismatch on the stress field at the crack tip, and the effect of work hardening mismatch on the fracture toughness. Zhao et al. [24,25] used the finite element model of the sandwich structure to analyze the difference in the crack tip field caused by the heterogeneity of yield strength in the heat-affected zone of the welded joint, and they discussed its influence on the driving force of yield strength mismatch crack propagation. Lee et al. [26,27] used the finite element model of sandwich structure to analyze the crack tip constraint and J-integral characteristics of the plastic hardening mismatch bi-material interface crack. Although the mechanical properties of actual welded joint materials vary greatly in different regions, the materials are continuous. Therefore, with the finite element model of sandwich structure, it is difficult to accurately characterize the uneven distribution of mechanical properties of welded joints because there will be discontinuities in the mechanical field near the material interface [28], which will affect the safety evaluation accuracy of local structures at the crack tip. To accurately characterize the non-heterogeneity of material mechanical properties in the finite element model, it is necessary to establish a finite element model with continuous changes in material mechanical properties. Xue et al. [29] used the user-defined material (UMAT) subroutine and user-defined field variable (USDFLD) subroutine methods to make up for the shortcomings of the sandwich welded joints finite element model, and the analysis results show that the UMAT subroutine can characterize the non-uniformity of mechanical properties of welded joints well. In our previous research [30], the continuous change model of material yield strength is established by the USDFLD subroutine in ABAQUS 6.14 software. This method has the advantage of accurately characterizing the continuous change of mechanical properties of welded joint materials and eliminating the discontinuity of the mechanical field in the sandwich structure finite element model. It is of great help to improve the accuracy of the structural safety evaluation of welded joints.

In ABAQUS 6.14, the user-defined field subroutine [31] can flexibly define the material properties. Li et al. [32] experimentally and numerically studied the static and fatigue failure of the cap beam-reinforced composite plate under a four-point bending load. The failure prediction model is established by using the USDFLD subroutine, and the numerical analysis of the cap beam-reinforced composite plate is carried out. The calculated predicted value is close to the experimental value, which shows that the USDFLD subroutine can be well-used to establish the material failure model. Based on the bearing capacity and shear transfer mechanism of the perfobond Leisten (PBL) shear connector of the conic curve, Wang et al. [33] adopted the development function of the user subroutine and the USDFLD subroutine, which effectively simulated the transverse shear expansion effect of the concrete dowel bar. The concrete damage plasticity (CDP) model has good robustness and stability, which has good convergence in the prediction and simulation of damage characteristics of concrete structures. Patrick et al. [34] realized the method modeling of ductile fracture through the USDFLD subroutine, which is used to simulate material fracture. Crack initiation and propagation are regarded as the characteristics of gradual loss of material integrity. The test results show that this method has good applicability. Ebadi-Rajoli et al. [35] established the relationship between damage parameters and the loading cycle through the USDFLD subroutine and calculated the damage accumulation to simulate the damage initiation and propagation of a composite interface layer under a cyclic load. Due to the complexity of strength and work hardening, there is a mismatch between the crack tip stress field and the fracture resistance in welded joints. A tensile crack is mainly caused by tensile stress, which leads to the material damage at the crack tip. This study simulates this process based on the maximum principal stress failure criterion. Therefore, the finite element model of uneven distribution of material ultimate strength in the maximum principal stress failure criterion is established by using the USDFLD subroutine to simulate the effect of uneven distribution of material ultimate strength on crack propagation and fracture resistance. The fracture toughness is calculated, and the effects of ultimate strength mismatch on the maximum principal stress field at the crack tip and crack propagation path are analyzed.

## 2. Materials and Methods

Based on the strain equivalence principle proposed by Lemaitre and Desmorat [36], this section will consider the coupling relationship between damage and elastic-plastic deformation by using equivalent stress instead of stress tensor in the elastic stress–strain relationship and von Mises yield criterion, to establish the coupling damage constitutive relationship. Under the assumption that the total strain can be expresssed as elastic strain and plastic strain:

(1) $$\varepsilon =\varepsilon^{e}+\varepsilon^{p}$$

In Formula (1), ε is strain tensor, $\varepsilon^{e}$ is elastic strain tensor and $\varepsilon^{p}$ is plastic strain tensor.

The elastic stress–strain response follows Hooke’s law:

(2) $$\sigma =(1-D)C:[\varepsilon -\varepsilon^{p}]$$

In Formula (2), σ is stress tensor, C is elastic tensor, and D is an isotropic damage variable.

The rate-dependent expression of stress tensor, σ, is:

(3) $$\dot{\sigma }=(1-D)C:[\dot{\varepsilon }-{\dot{\varepsilon }}^{p}]-\frac{\sigma }{1-D}\dot{D}$$

In Equation (3), $\dot{\sigma }$ is stress rate tensor, $\dot{\varepsilon }$ is strain rate tensor, ${\dot{\varepsilon }}^{p}$ is equivalent plastic strain rate tensor, and is the damage variable rate.

Equation (4) represents the Von Mises yield condition:

(4) $$F_{y}=\sqrt{\frac{3}{2}(\tilde{s}-\tilde{a}):(\tilde{s}-\tilde{a})}-\sigma_{y}\leq 0$$

In Equation (4), $\sigma_{y}$ is the radius of yield surface, $\tilde{s}$ is deviatoric stress tensor of damaged materials, and $\tilde{a}$ is back stress tensor of damaged materials. $\tilde{s}$ and $\tilde{a}$ are defined as Equations (5) and (6), respectively:

(5) $$\tilde{s}=s/(1-D)$$

(6) $$\tilde{a}=a/(1-D)$$

In Equation (5), s is stress tensor. In Equation (6), a is back stress tensor.

In this research, the orthogonal plastic flow criterion is adopted, which is expressed as Equation (7):

(7) $${\dot{\varepsilon }}^{p}=\sqrt{\frac{3}{2}}\dot{p}n$$

In Equation (7), $\dot{p}$ is equivalent plastic strain rate, and n is unit direction tensor, which can be calculated according to Equation (8):

(8) $$n=\frac{\tilde{s}-\tilde{a}}{\left\|\tilde{s}-\tilde{a}\right\|}$$

For the criterion of material damage, it is considered that when *D* = 1, the material fails, and when *D* = 0, the element is in the nondestructive state, and *D* = *D_c_* means the element is in a critical state of fracture. The maximum principal stress failure criterion is used in this study.

(9) $$\{\begin{array}{c} D=0\\ D=D_{c} \end{array}\begin{array}{c} ,\\ , \end{array}\begin{array}{c} \mathrm{if}\;\sigma_{\max\;}<\sigma_{b}\\ \mathrm{if}\;\sigma_{\max\;}\geq \sigma_{b} \end{array}$$

In Equation (9), $\sigma_{\max\;}$ is maximum principal stress, and $\sigma_{b}$ is ultimate strength. When the maximum principal stress is greater than the ultimate strength, the damage variable is equal to the critical damage variable, and the element is in the critical state of failure. The ultimate strength is related to the field variable, θ, and its expression is as follows:

(10) $$\sigma_{b}(\theta ,\varepsilon )$$

The field variables, θ, in Equation (10) are related to the geometry coordinates.

(11) θ=ψ(x,y)

where ψ(x,y) is a function related to the spatial position of geometry in Equation (11), and the subroutine is used to establish the relationship function between field variables and the spatial position. The material model of uneven distribution of ultimate strength can be realized by applying it to the calculation of ABAQUS 6.14 finite element software.

## 3. Numerical Simulation Procedures

**Figure 1:**
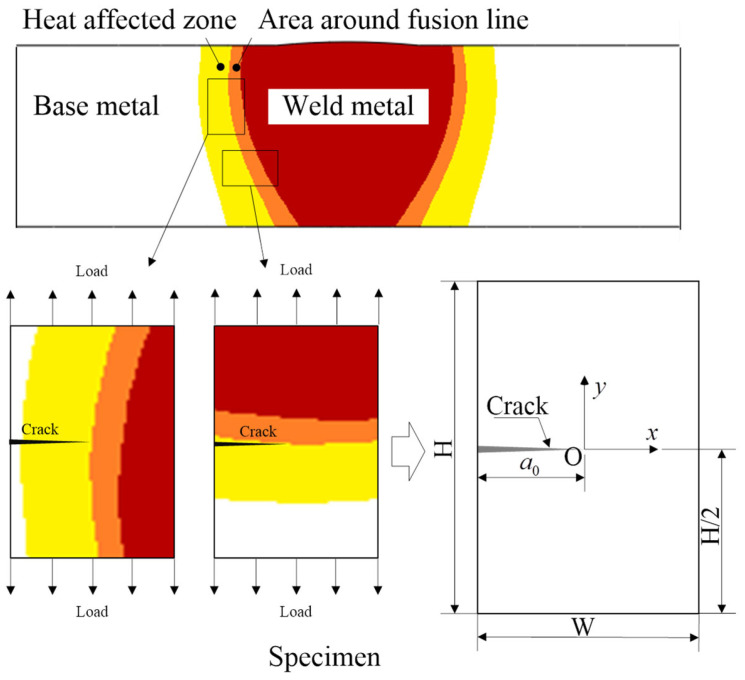
The dimensions of welded joints.

To explore the influence of local heterogeneity of ultimate strength on crack propagation resistance, the finite element model of welded joints was simplified, and the following three assumptions were made: (1) the mechanical properties of base metal and welding material are homogeneous, (2) ignore the influence of welding residual stress, and (3) continuous linear distribution of material ultimate strength in the bi-material connection area. The size, initial crack location, and material distribution of the simplified welded joint are shown in Figure 1. The welded joint is composed of three parts: base metal, weld material, and a dual-material connection area composed of an area around fusion line and a heat-affected zone at the connection between the base metal and weld material. The height of the welded joint was H = 15 mm, and the width was W = 10 mm. Initial crack length was *a_0_* = 5 mm. The origin of the spatial coordinate system of the model is located at the initial crack tip. The crack propagation direction is the x-axis direction.

**Figure 2:**
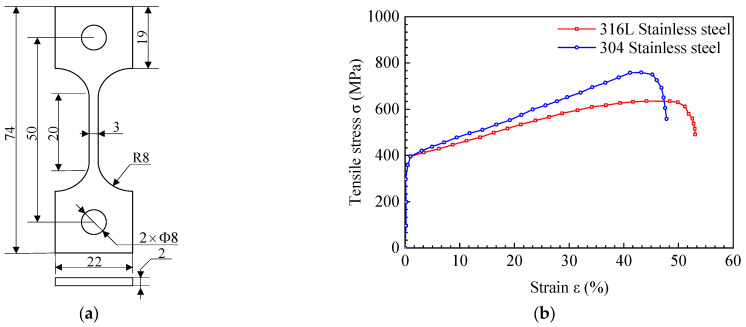
Stress–strain tensile test. (**a**) Tensile test size, and (**b**) stress–strain curve of 304 and 316 L stainless-steel.

The material property is shown in Figure 2b. The stress–strain data of 304 and 316 L stainless-steel were obtained though tensile experiments, the sample sizes of which are shown in Figure 2a. The tensile experiments’ results show that the ultimate strength of 304 stainless-steel was *σ_b,304_* = 700 MPa, and that of 316 L stainless-steel was *σ_b,316L_* = 600 MPa. The material property of the finite element model of the base metal and the welded metal was set according to these two materials. The elasticity modulus of the weldment was E = 210 GPa, Poisson’s ratio was *μ* = 0.3, and the yield strength was *σ_y_* = 400 MPa. The material ultimate strength distribution in each area of the finite element model of the welded joint is shown in Table 1.

**Figure 3:**
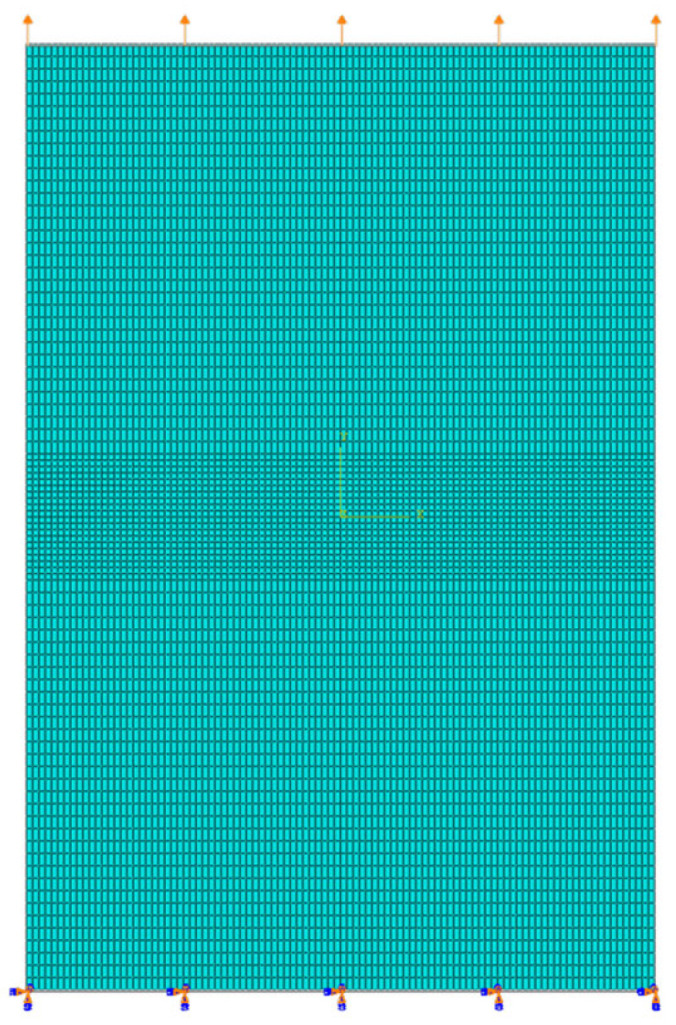
Mesh and elements.

**Figure 4:**
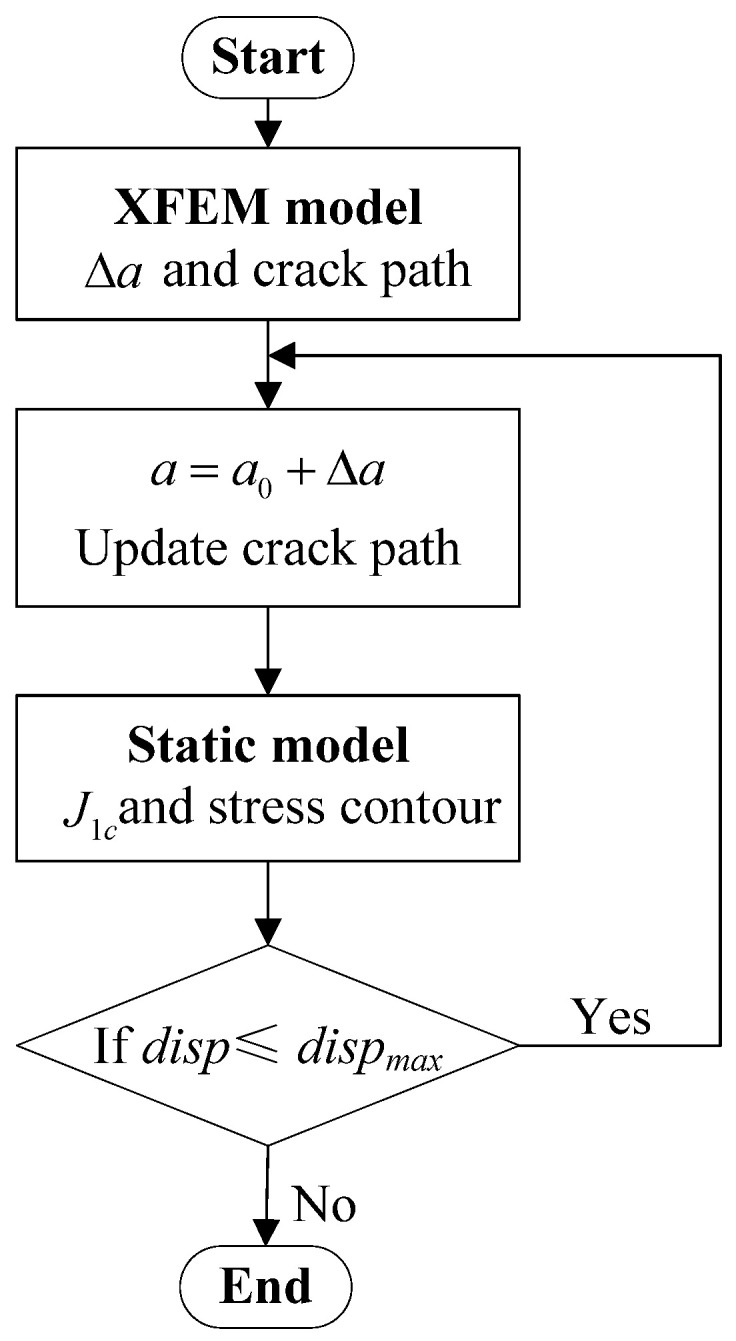
Calculation process.

The bottom of the welded joint finite element model is fixed, and the displacement load is applied at the top of the welded joint finite element model; thus, the flat plate receives uniform tensile stress. The displacement load is *disp_max_*= 0.08 mm, which is set in ABAQUS 6.14. After finite element meshing, as shown in Figure 3, the number of elements was 8600. The element type is CPE4R. In the process of the numerical simulation, the calculation flow chart is as shown in Figure 4. Firstly, the crack propagation path, crack propagation increment, Δa, and critical failure displacement load were calculated when the element began to break through the extended finite element method (XFEM). Secondly, according to the crack growth path and the crack growth increment, Δa, extracted, a static crack model without crack growth was established. The critical failure displacement load was used as the boundary condition to calculate the J-integral under the critical load state of the welded joint. The J-integral is the critical fracture resistance of the material in fracture and forward propagation [37], which is used as the critical fracture toughness, *J_1c_*. This process is cycled until the displacement load reaches the set maximum value, *disp _max_*. Thus, the fracture toughness of materials in different regions during crack propagation was obtained.

## 4. Results and Discussion

### 4.1. The Results of Ultimate Strength Change in y-Direction

**Figure 5:**
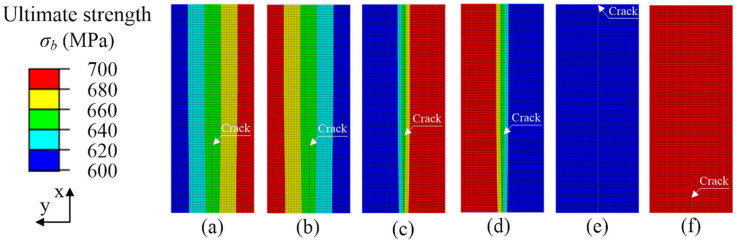
Crack propagation paths and ultimate strength distribution with mismatch coefficients in the y-direction. (**a**) *G_y_* = −50 MPa/mm, (**b**) *G_y_* = 50 MPa/mm, (**c**) *G_y_* = −100 MPa/mm, (**d**) *G_y_* = 100 MPa, (**e**) σ*_b,316L_* = 600 MPa, (**f**) σ*_b,304_* = 700 MPa.

The crack propagation path in the case of different ultimate strength mismatches in the y-direction at the interface of the two materials is shown in Figure 5. The cloud diagram shows the distribution of material ultimate strength. The crack propagation path in materials with different ultimate strengths can be seen. Since the material ultimate strengths on both sides of the crack are different, the mismatch of the ultimate strengths on both sides of the crack leads to the deflection of the crack propagation direction. As shown in Figure 5a–d, the ultimate strength change gradient *G_y_* > 0 and *G_y_* < 0. In the process of crack deflection, it always deflects to the side of low ultimate strength material. The greater the ultimate strength change gradient, the greater the crack deflection. Figure 5e,f show the homogeneous material crack propagation path, with ultimate strength of σ*_b,316L_* = 600 MPa and σ*_b,304_* = 700 MPa, respectively, and the ultimate strengths on both sides of the crack are the same. The crack propagation path is a straight line. In addition, for the material with high ultimate strength, the crack propagation length was smaller, as shown in Figure 5e, while for the material with low ultimate strength, the crack penetrated the specimen completely, as shown in Figure 5f.

**Figure 6:**
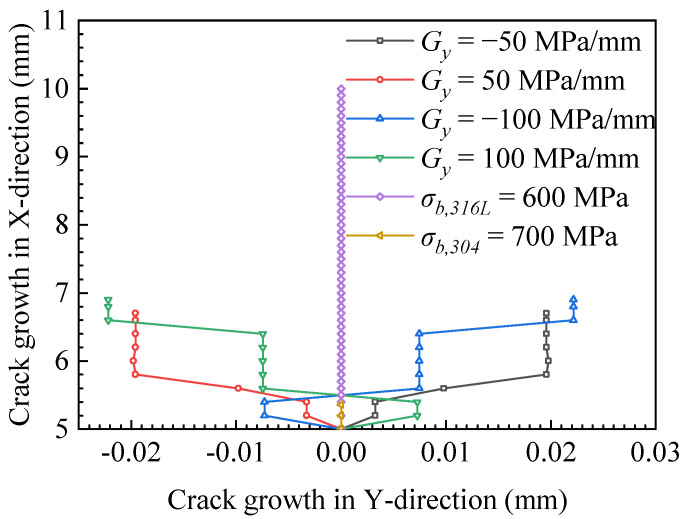
Crack propagation paths with different ultimate strength mismatch coefficients in the y-direction.

To clearly show the crack propagation length under different ultimate strength mismatches, the crack propagation path in Figure 5 was extracted, and the crack propagation path diagram in Figure 6 was drawn. In Figure 6, the ordinate represents the projection of the welded joint in the x-direction. The abscissa represents the deflection of the crack in the y-direction of the welded joint. Figure 6 clearly shows that the mismatch of ultimate strength leads to the deviation of the crack propagation path. The crack deflection angle of the gradient |*G_y_*|= 50 MPa/mm was 0.1606°, and the crack length was 6.85 mm. The crack deflection angle of the gradient |*G_y_*|= 100 MPa/mm was 0.1814°, and the crack length was 6.95 mm. The gradient of ultimate strength increased from |*G_y_*|= 50 to |*G_y_*|= 100 MPa/mm, the crack deflection angle increased by 0.018%, and the crack length increased by 1.46%. When the change gradient was *G_y_* = ±50 MPa/mm, the crack deflection was less than that of the change gradient of *G_y_* = ±100 MPa/mm. Therefore, the ultimate strength mismatch caused the crack to expand in the low ultimate strength direction. The larger the ultimate strength change gradient is, the larger the crack deflection angle will be. When the material is homogeneous, the ultimate strength of the two materials on both sides of the crack is the same. The crack propagates in a straight line. The low ultimate strength material (σ*_b,316L_* = 600 MPa) showed crack penetration at the welded joint, but the high ultimate strength material (σ*_b,304_* = 700 MPa) had the shortest crack propagation length.

**Figure 7:**
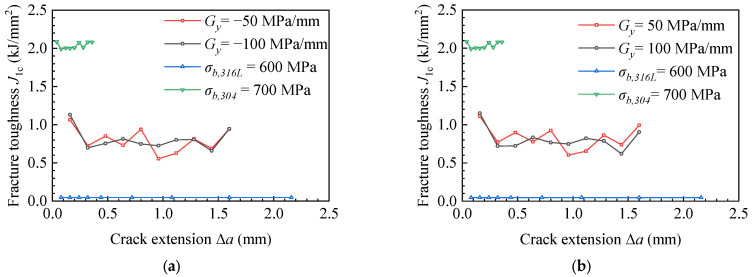
Fracture toughness during crack propagation with different ultimate strength mismatch coefficients in the y-direction. (**a**) *G_y_* < 0 and (**b**) *G_y_* > 0.

Fracture toughness refers to the impedance value displayed by the material when there are cracks or crack-like defects in the sample or component, which is no longer rapid fracture with the increase of load. Such fracture toughness value can be expressed by a single parameter describing the mechanical state of the crack tip, such as energy release rate, G, stress intensity factor, K, crack tip opening displacement (CTOD), and J-integral. In this research, we used the critical J-integral when the material near the crack failed as the evaluation parameter of fracture toughness, which is called critical fracture toughness here. Figure 7 shows the change of critical fracture toughness during crack propagation. To increase the comparison, the fracture toughness curves of the homogeneous material, for which the ultimate strengths are σ*_b,316L_* = 600 MPa and σ*_b,304_* = 700 MPa, were added in Figure 7. The fracture toughness values of homogeneous materials of 304 and 316 L obtained from the experiment were *J_1__c__,304_exp_* = 2.011 kJ/mm^2^ and *J_1c,316L_exp_* = 0.047 kJ/mm^2^. The average values of finite element calculation results were *J_1c,304_ave_* = 2.038 kJ/mm^2^ and *J_1c,316L_ave_* = 0.046 kJ/mm^2^, respectively. The calculation error of fracture toughness of 304 stainless-steel was 1.34%, and that of 316 L stainless-steel was 2.13%, and the calculations are in agreement with the experiment results in Figure 7a,b, which verifies the calculation model. As can be seen in Figure 7a,b, when the ultimate strength change gradients were *G_y_* < 0 and *G_y_* > 0, the fracture toughness showed a downward trend during crack propagation. The material with low ultimate strength had low fracture toughness, resulting in deflection during crack propagation, as shown in Figure 5a–d. In Figure 7, the fracture toughness of homogeneous materials is approximately a horizontal straight line, indicating that the fracture toughness of homogeneous materials is constant. The material with high ultimate strength had high fracture toughness, which led to a small crack propagation length under the same load, as shown in Figure 5f. The material with low ultimate strength had small fracture toughness and a long crack propagation length, as shown in Figure 6 and Figure 7a,b (The curve of σ*_b,316L_* = 600 MPa). In addition, in Figure 7, the fracture toughness curves of ultimate strength variation gradients *G_y_* < 0 and *G_y_* > 0 are located in homogeneous materials’ (σ*_b,316L_* = 600 MPa, σ*_b,304_* = 700 MPa) fracture toughness curves, so under the same load condition, the crack length in the material with ultimate strength change gradients *G_y_* < 0 and *G_y_* > 0 was between the length of homogeneous materials σ*_b,316L_*= 600 MPa and σ*_b,304_* = 700 MPa, as shown in Figure 6.

**Figure 8:**
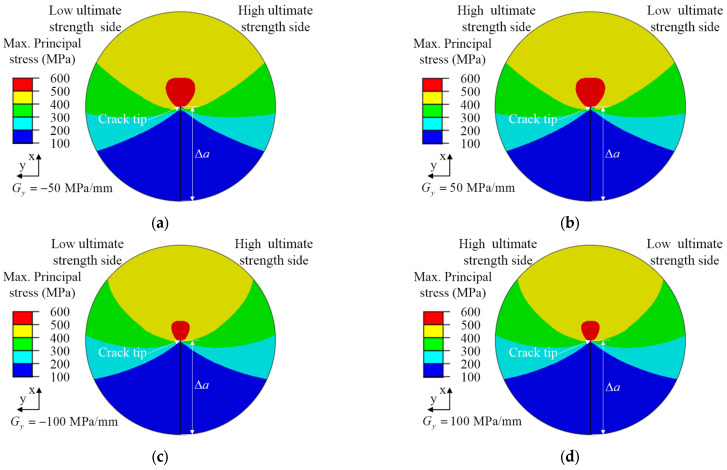

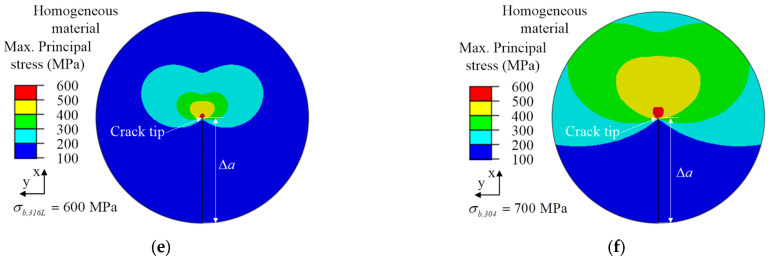
Maximum principal stress field of crack tip with different ultimate strength mismatch coefficients of crack propagation length Δa=0.6 mm in the y-direction. (**a**) *G_y_* = −50 MPa/mm, (**b**) *G_y_* = 50 MPa/mm, (**c**) *G_y_* = −100 MPa/mm, (**d**) *G_y_* = 100 MPa/mm, (**e**) σ*_b,316L_* = 600 MPa, (**f**) σ*_b,304_* = 700 MPa.

Figure 8 shows the contour of the maximum principal stress at the crack tip with the crack propagation length Δa=0.6 mm under the same geometric constraints. It is shown that the ultimate strength mismatch between 304 and 316 L stainless-steel will affect the distribution and value of the maximum principal stress before the crack tip. When the ultimate strength change gradients were *G_y_* < 0 and *G_y_* > 0, the ultimate strength mismatch existed on both sides of the crack, and the maximum principal stress field at the crack tip deflected slightly to the direction of the low ultimate strength side. The larger the ultimate strength change gradient, the smaller the distribution range of the maximum principal stress at the crack tip, as shown in Figure 8a–d. For homogeneous materials, the mechanical properties of materials on both sides of the crack are the same, so the maximum principal stress at the crack tip is symmetrically distributed, as shown in Figure 8e,f.

In general, the above results show that for interface cracks with different ultimate strength mismatches, the change of fracture toughness is the material constraint effect caused by local cracks. For the materials with ultimate strength change gradients *G_y_* < 0 and *G_y_* > 0, their fracture toughness is between the highest ultimate strength and the lowest ultimate strength. Therefore, the ultimate strength mismatch of materials has a harmful effect on the weldment.

### 4.2. The Results of Ultimate Strength Change in x-Direction

**Figure 9:**
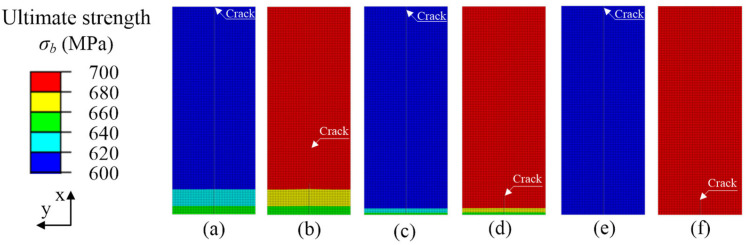
Crack propagation paths and ultimate strength distribution with mismatch coefficients in the x-direction. (**a**) *G_x_* = −50 MPa/mm, (**b**) *G_x_* = 50 MPa/mm, (**c**) *G_x_* = −100 MPa/mm, (**d**) *G_x_* = 100 MPa/mm, (**e**) σ*_b,316L_* = 600 MPa, (**f**) σ*_b,304_* = 700 MPa.

**Figure 10:**
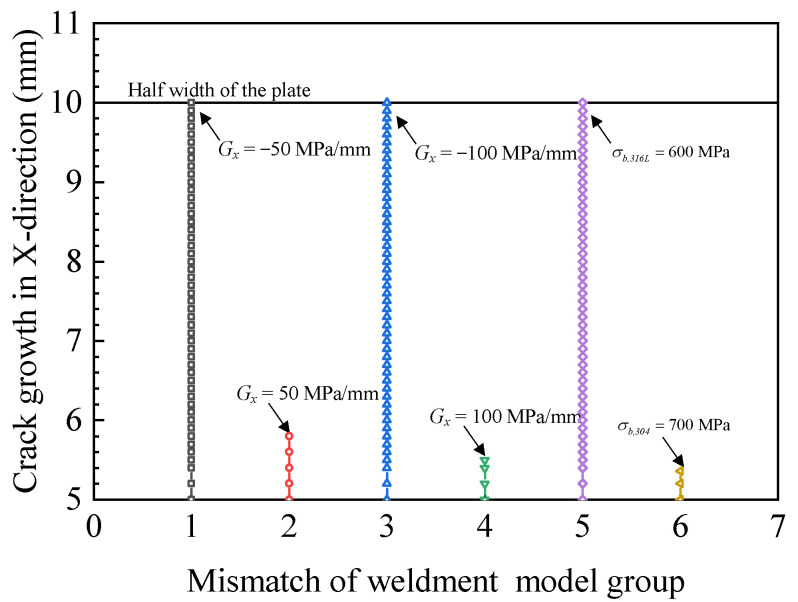
Crack propagation lengths of different ultimate strength mismatch coefficients in the x-direction.

Figure 9 shows the crack propagation path in the case of different ultimate strength mismatches in the x-direction at the interface of the two materials. The contour shows the distribution of material ultimate strength and the crack propagation path. Since the change direction of material ultimate strength was the same as that of the crack propagation direction, the material ultimate strengths on both sides of the crack were the same, so the crack did not deflect and propagated along a straight line. To clearly show the crack propagation length under different ultimate strength mismatches, the crack propagation path data in Figure 9 were extracted and the crack propagation length diagram in Figure 10 was obtained. Figure 9a shows that the crack propagated in the material with the ultimate strength change gradient *G_x_* = −50 MPa/mm, as well as the ultimate strength change gradient *G_x_* = −100 MPa/mm, as shown in Figure 9c. The crack propagated from the material at the high ultimate strength side to the material at the low ultimate strength side. The crack completely penetrated the specimen, as shown in Figure 10. However, the displacement load of crack penetration in the material with ultimate strength change gradient *G_x_* = −50 MPa/mm was 0.456 mm, while the displacement load of crack penetration in the material with ultimate strength change gradient *G_x_* = −100 MPa/mm was 0.312 mm. Figure 9b,d show the ultimate strength change gradients *G_x_* = 50 and *G_x_* = 100 MPa/mm, respectively. In the process of crack propagation from the low ultimate strength material to the high ultimate strength material, the crack propagation distance was small. The greater the gradient of the ultimate strength change, the shorter the crack length, as shown in Figure 10. Figure 9e,f show the homogeneous materials’ (σ*_b,316L_* = 600 MPa and σ*_b,304_* = 700 MPa) crack propagation path, and it can be found that in the material with high ultimate strength, the crack propagation length was smaller, while in the material with low ultimate strength, the crack completely penetrated the specimen. The displacement load reached 0.0216 mm when the crack penetrated the specimen.

**Figure 11:**
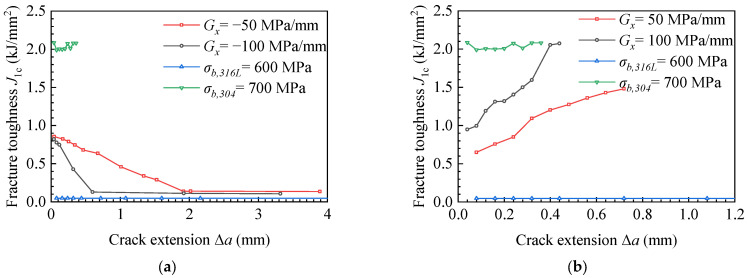
Fracture toughness during crack propagation with different ultimate strength mismatch coefficients in the y-direction. (**a**) *G_x_* < 0 and (**b**) *G_x_* > 0.

Figure 11 presents the fracture toughness during crack propagation with different ultimate strength mismatch coefficients in the x-direction. The fracture toughness was the same as that of 304 and 316 L materials in Figure 7, and the fracture toughness values of 304 and 316 L homogeneous materials obtained from the experiment were *J_1__c__,304_* = 2.011 kJ/mm^2^ and *J_1c,316L_* = 0.042 kJ/mm^2^. The experimental data were in agreement with the average values of the finite element calculation results in Figure 11a,b, which verifies the calculation model. As the gradient of ultimate strength increased from *G_x_* = 50 to *G_x_* = 100 MPa/mm, the crack length was reduced from 5.8 to 5.5 mm, which decreased by 5.17%. It can be found from Figure 11a that in the materials with ultimate strength change gradients *G_x_* = −50 and *G_x_* = −100 MPa/mm, the crack expanded into the low ultimate strength material side, as shown in Figure 9a,c. The lower ultimate strength led to a lower fracture toughness of the material, so the fracture toughness showed a downward trend in the process of crack propagation.

The fracture toughness was constant after the crack passed through the bi-material connection area and entered the weld material area. In Figure 11b, for the materials with ultimate strength change gradients *G_x_* = −50 and *G_x_* = −100 MPa/mm, the crack expanded into the high ultimate strength material, as shown in Figure 9b,d. As the gradient of ultimate strength increased from G_x_ = 50 to G_x_ = 100 MPa/mm, the slope of the fracture toughness curve increased from 1.33 to 2.57 (kJ/mm^2^)/mm, which increased by 51.63%. On the contrary, when the ultimate strength gradient increased from *G_x_* = −100 to *G_x_* = −50 MPa/mm, the slope of the fracture toughness curve decreased from 1.13 to 0.38 (kJ/mm^2^)/mm, which decreased by 51.01%. The materials with higher ultimate strength had higher fracture toughness, so the fracture toughness increased in the process of crack propagation. For homogeneous materials (σ*_b,316L_* = 600 MPa, σ*_b,304_* = 700 MPa), the ultimate strength did not change during crack propagation. As shown in Figure 9e,f, the fracture toughness curve in Figure 11a,b is approximately a horizontal straight line, indicating that the fracture toughness of homogeneous material is constant.

**Figure 12:**
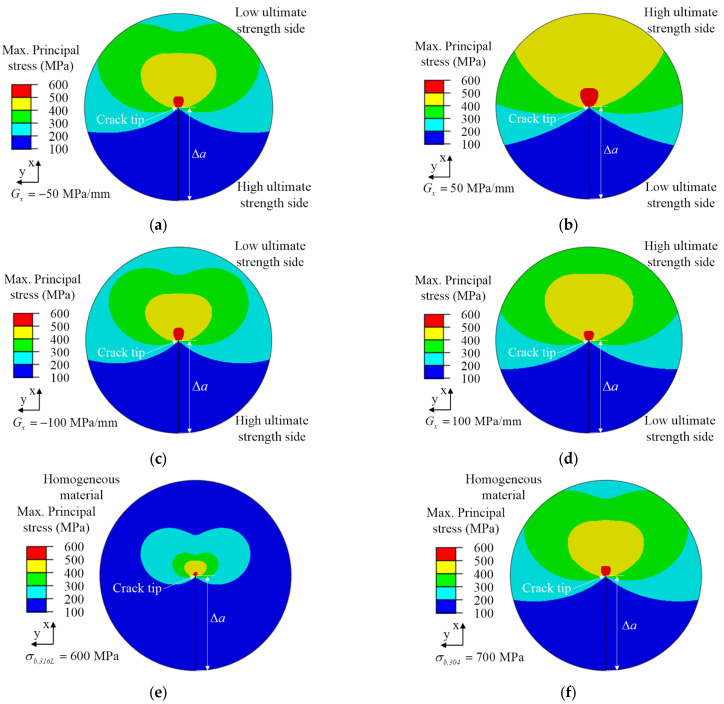
Maximum principal stress field of the crack tip with different ultimate strength mismatch coefficients of crack propagation length Δa=0.6 mm in the x-direction. (**a**) *G_x_* = −50 MPa/mm, (**b**) *G_x_* = 50 MPa/mm, (**c**) *G_x_* = −100 MPa/mm, (**d**) *G_x_* = 100 MPa/mm, (**e**) σ*_b,316L_* = 600 MPa, (**f**) σ*_b,304_* = 700 MPa.

Figure 12 shows the crack growth length, Δa=0.6 mm, with different ultimate strength mismatch coefficients in the x-direction for the maximum principal stress field at the crack tip under the same geometric constraints. It can be found that the change gradient of ultimate strength will affect the distribution and value of the maximum principal stress in front of the crack tip. From the maximum principal stress field at the crack tip in Figure 12, it can be found that the maximum principal stress at the crack tip was symmetrically distributed, because the crack propagation direction was the same as the change direction of the material ultimate strength and the material mechanical properties on both sides of the crack were the same. Comparing the maximum principal stress field at the crack tip with the ultimate strength change gradient *G_x_* < 0 as shown in Figure 12a,c, and the maximum principal stress field at the crack tip with the ultimate strength change gradient *G_x_* > 0 as shown in Figure 12b,d, it can be found that when the crack propagated from the high ultimate strength material side to the low ultimate strength material side, the crack tip pointed to the material with lower ultimate strength, which was caused by the ultimate strength mismatch of the materials. The material with low ultimate strength had little constraint effect on the maximum principal stress at the crack tip, so the distribution range of the maximum principal stress at the crack tip was small, as shown in Figure 12a,c. On the contrary, the distribution range of the maximum principal stress at the crack tip was large, as shown in Figure 12b,d. For homogeneous materials, the crack tip restraint effect of materials with high ultimate strength was larger and that of materials with low ultimate strength was smaller. Therefore, Figure 12e shows the distribution range of the maximum principal stress at the crack tip, of which the ultimate strength of σ*_b,316L_* = 600 MPa was less than the ultimate strength of σ*_b,304_* = 700 MPa.

The analysis results showed that for different ultimate strength change gradients, the local material ultimate strength mismatch at the crack tip affects the material fracture toughness. The material constraint effect caused by the mismatch of ultimate strength influenced the distribution of the maximum principal stress field at the crack tip. For the crack propagation process in materials with ultimate strength change gradient *G_x_* < 0, their fracture toughness gradually decreased. Therefore, the material restraint effect caused by the interaction between the two materials is hazardous to the fracture resistance of the material.

## 5. Conclusions and Observations

Based on the numerical analysis of the maximum principal stress failure criterion model, the numerical analysis of the effect of ultimate strength mismatch on the fracture resistance of two kinds of cracks (the change of the crack propagation direction was the same as the ultimate strength, and the crack propagation direction was perpendicular to the ultimate strength) was studied. The main results obtained from the simulation study of this work are summarized as follows:

1. When the crack propagation direction was perpendicular to the change direction of ultimate strength, the crack path as well as the maximum principal stress field at the crack tip always deflected to the side of low ultimate strength. The fracture toughness decreased slightly during crack propagation.
2. The crack length in the process of crack propagation to the low ultimate strength material side was greater than that to the high ultimate strength material, but the crack propagation resistance as well as the area of the maximum principal stress caused by material constraints were less than that to the low ultimate strength material, when the crack propagation direction was the same as the change direction of ultimate strength.
3. When evaluating the structural safety of cracks in the connection area, where the ultimate strength of dissimilar metal-welded joints is between the ultimate strength of the base metal and the weld material, only considering the ultimate strength of the base metal or the weld material will inevitably lead to a conservative estimation or unsafe estimation results. Therefore, it is recommended to use the fracture toughness of local areas for evaluation.

## Figures and Tables

**Table 1:** Material ultimate strength of welded joint finite element model.

Direction	Gradient Factor, *G* (MPa/mm)	Weldment Component	$\mathrm{Ultimate}\;\mathrm{Strength},\;\sigma_{b}\;(\mathrm{MPa})$	Distribution (mm)
*x*-direction	*G_x_* = −50	Base metal (304)	700	−5 ≤ x < −1
		Fusion metal	−50x + 650	−1 ≤ x ≤ 1
		Weld metal (316 L)	600	1 < x ≤ 5
	*G_x_* = 50	Base metal (316 L)	600	−5 ≤ x < −1
		Fusion metal	50x + 650	−1 ≤ x ≤ 1
		Weld metal (304)	700	1 < x ≤ 5
	*G_x_* = −100	Base metal (304)	700	−5 ≤ x < −0.5
		Fusion metal	100x + 650	−0.5 ≤ x ≤ 0.5
		Weld metal (316 L)	600	0.5 < x ≤ 5
	*G_x_* = 100	Base metal (316 L)	600	−5 ≤ x < −0.5
		Fusion metal	−100x + 650	−0.5 ≤ x ≤ 0.5
		Weld metal (304)	700	0.5 < x ≤ 5
*y*-direction	*G_y_* = −50	Base metal (304)	700	−7.5 ≤ y < −1
		Fusion metal	−50y + 650	−1 ≤ y ≤ 1
		Weld metal (316 L)	600	1 < y ≤ 7.5
	*G_y_* = 50	Base metal (316 L)	600	−7.5 ≤ y < −1
		Fusion metal	50y + 650	−1 ≤ y ≤ 1
		Weld metal (304)	700	1 < y ≤ 7.5
	*G_y_* = −100	Base metal (304)	700	−7.5 ≤ y < −0.5
		Fusion metal	−100y + 650	−0.5 ≤ y ≤ 0.5
		Weld metal (316 L)	600	0.5 < y ≤ 7.5
	*G_y_* = 100	Base metal (316 L)	600	−7.5 ≤ y < −0.5
		Fusion metal	100y + 650	−0.5 ≤ y ≤ 0.5
		Weld metal (304)	700	0.5 < y ≤ 7.5
Homogeneous material	-	316 L	600	-
	-	304	700	-

## Data Availability

The data presented in this study are available upon request from the corresponding author.

## Nomenclature

ε	strain tensor	$\sigma_{b}$	ultimate strength
$\varepsilon^{e}$	elastic strain tensor	$D_{c}$	critical damage variable
$\varepsilon^{p}$	plastic strain tensor	θ	field variable
σ	stress tensor	ψ	field variable distribution function
D	isotropic damage variable	x	x coordinate value
C	elastic tensor	y	y coordinate value
$\dot{\sigma }$	stress rate	E	elasticity modulus
$\dot{D}$	damage variable rate	*μ*	Poisson’s ratio
$\dot{\varepsilon }$	strain rate	*a_0_*	initial crack length
${\dot{\varepsilon }}^{p}$	equivalent plastic strain rate	H	width of welded joint
$F_{y}$	Von Mises yield condition	W	height of welded joint
$\sigma_{y}$	radius of yield surface	Δa	crack growth increment
$\tilde{s}$	partial stress tensor of damaged materials	*σ_b,316L_*	ultimate strength of 316L stainless-steel
$\tilde{a}$	back stress tensor of damaged materials	*σ_b,304_*	ultimate strength of 304 stainless-steel
s	stress tensor	*J_1c_*	critical fracture toughness
a	back stress tensor	*disp_max_*	the max displacement load
$\dot{p}$	equivalent plastic strain rate	*disp*	displacement load
n	unit direction tensor	*G_x_*	gradient factor in *x*-direction
$\sigma_{\max}$	maximum principal stress	*G_y_*	gradient factor in *y*-direction
